# EMBRACING DIVERSITY BY FOSTERING STRONG COMMUNITY CONNECTIONS

**DOI:** 10.1093/geroni/igac059.548

**Published:** 2022-12-20

**Authors:** Robyn Golden, Erin Emery-Tiburcio, Rani Snyder

## Abstract

Age-Friendly Health Systems are essential; however, most health and healing take place outside of the walls of a hospital or physician’s office. Thus, it is imperative that health systems foster strong community connections, in particular with older adults from underserved communities who experience multiple barriers to quality, age-friendly health care. At Rush, there are several interdisciplinary initiatives designed to ensure that the 4Ms reach beyond the medical center and impact historically marginalized communities. In this symposium, we present three of these innovative approaches to bringing the 4Ms to into the community. Each presentation will describe the program and community served, explore how the 4Ms concepts are integrated, and present outcomes data on the program impact to date. Facilitating Caregiver Health and Wellness: Age-Friendly Health System Caring for Caregivers (AFHS-C4C) is an initiative to expand the age friendly health system to include caregivers. CATCH-ON Connect enables older adults to do What Matters to them by expanding virtual access to older adults, often from underserved communities. Finally, Rush@Home addresses the 4Ms by removing barriers to care and enabling older adults with medically complex needs to receive care in their home. The interactions of these programs will be discussed briefly by the session chair, along with service and policy implications of multiple programs addressing the 4Ms of an Age-Friendly Health System within one community.

